# Supplementary material for: Common and Rare PCSK9 Variants Associated with Low-Density Lipoprotein Cholesterol Levels and the Risk of Diabetes Mellitus: A Mendelian Randomization Study
Source: Int J Mol Sci. 2022 Sep 8;23(18):10418. doi: 10.3390/ijms231810418 (PMC9499600; doi:10.3390/ijms231810418)
Supplement: Supplementary file 1 [file ijms-23-10418-s001.zip › Supplementary_20220829 final.pdf]

Supplementary Method S1

Definitions of hypertension, diabetes mellitus, obesity, current smoking, microalbuminuria and metabolic syndrome

Hypertension was defined as systolic blood pressure (BP) of ≥140 mmHg, diastolic BP of ≥90 mmHg or a self-reported history of hypertension. Diabetes mellitus (DM) was defined as a fasting plasma glucose level of ≥126 mg/dL, a glycohemoglobin value of ≥6.5%, or a self-reported history of DM. Obesity was defined as a BMI of ≥25 kg/m^2. Current smoking was defined as regular cigarette smoking at the time of survey. Because of the absence of urine creatinine level, only spot urine albumin level was used for the evaluation of urine albumin. Microalbuminuria was defined as urine albumin of ≥30 mg/L.

Because medication histories were unavailable, metabolic syndrome characteristics were based on the recent update of the third report of the National Cholesterol Education Program's Adult Treatment Panel III criteria [1] with modifications. Participants with three or more of the following attributes are typically defined as having metabolic syndrome: (1) BP of ≥130/85 mmHg or a history of hypertension; (2) triglyceride level of ≥150 mg/dL; (3) high-density lipoprotein cholesterol level of <40 mg/dL for men or <50 mg/dL for women; (4) fasting plasma glucose of ≥100 mg/dL or a history of DM; and (5) waist circumference of >90 cm for men or >80 cm for women.

Reference:

1. Grundy SM, Cleeman JI, Daniels SR, Donato KA, Eckel RH, Franklin BA, et al. Diagnosis and management of the metabolic syndrome: an American Heart Association/National Heart, Lung, and Blood Institute Scientific Statement. Circulation. 2005; 11: 2735–52.

Supplementary Method S2

Sensitivity analysis for causal inference from standard Mendelian randomization (MR) with multiple genetic variants

We first performed multivariate analysis to avoid the effect of measurable confounders. Funnel plots of the instrumental variable (IV) precisions against the IV estimates for each genetic variant were then plotted to estimate the directional pleiotropy. The Inverse-variance weighted (IVW) method (Burgess et al., 2013), which involves using a formula from the meta-analysis literature (Johnson et al., 2012), that combines the ratio estimates from each uncorrelated genetic variants into an overall estimate, is asymptotically equal to the two stage least square (2SLS) estimate commonly used with individual-level data. The IVW method remains unbiased as the number of single nucleotide variation (SNV) increases. The corrected standard error can be obtained by fitting a random effect of the IVW model (Bowden et al., 2017). The IVW estimate is an efficient analysis method when all genetic variants are valid IVs, whereas a simple median estimator provides a consistent estimate of the causal effect when less than 50% of the studied genetic variants are valid (Bowden et al., 2016). IVW regression was performed using the analysis software Meta-Essentials_1.4 from: www.erim.eur.nl/research-support /meta-essentials (Suurmond et al., 2017). When the individual estimates vary considerably, the simple median estimator is insufficient. The weighted median uses the median of the weighted ratio with standardized weights so that the sum of the weights is 1. The weighted median provides a consistent estimate if at least 50% of the weight comes from valid IVs. Both types of median regression were performed using SPSS 22 statistics software (SPSS Inc, Chicago, IL). The MR-Egger regression method was derived from the meta-analysis literature (Egger et al., 1997). This method is used for the assessment of small-study bias and to detect the overall directional pleiotropy from separate genetic variants if an intercept term differs from zero (Bowden et al., 2016). The MR-Egger regression method provides an estimate for the true causal effect that is consistent even if all genetic variants are invalid for the IV assumption (Bowden et al., 2016). We applied this method by using the analysis software Meta-Essentials_1.4 and SPSS.

Reference:

1. Bowden, J., Davey Smith, G., Haycock, P. C., & Burgess, S. 2016. Consistent

Estimation in Mendelian Randomization with Some Invalid Instruments Using a Weighted Median Estimator. Genet Epidemiol, 40(4): 304-314.

2. Bowden, J., Del Greco, M. F., Minelli, C., Davey Smith, G., Sheehan, N., & Thompson, J. 2017. A framework for the investigation of pleiotropy in two-sample summary data Mendelian randomization. Stat Med, 36(11): 1783-1802.
3. Burgess, S., Bowden, J., Fall, T., Ingelsson, E., & Thompson, S. G. 2017. Sensitivity Analyses for Robust Causal Inference from Mendelian Randomization Analyses with Multiple Genetic Variants. Epidemiology, 28(1): 30-42.
4. Burgess, S., Butterworth, A., & Thompson, S. G. 2013. Mendelian randomization analysis with multiple genetic variants using summarized data. Genet Epidemiol, 37(7): 658-665.
5. Egger, M., Davey Smith, G., Schneider, M., & Minder, C. 1997. Bias in meta-analysis detected by a simple, graphical test. BMJ, 315(7109): 629-634.
6. Johnson, T., & Uk, S. 2012. Efficient calculation for multi-SNP genetic risk scores.
7. Palmer, T. M., Lawlor, D. A., Harbord, R. M., Sheehan, N. A., Tobias, J. H., Timpson, N. J., et al. 2012. Using multiple genetic variants as instrumental variables for modifiable risk factors. Stat Methods Med Res, 21(3): 223-242.
8. Suurmond, R., van Rhee, H., & Hak, T. 2017. Introduction, comparison, and validation of Meta-Essentials: A free and simple tool for meta-analysis. Res Synth Methods, 8(4): 537-553.

Supplementary Table S1. Baseline characteristics of study subjects according to sex.

Clinical and laboratory parameters	Male	Female	*p* value
Number	26,797	48,644	
Anthropology			
Age (years)	50.0 (40.0 - 59.0)	50.0 (41.0 - 58.0)	0.4973
Waist circumference (cm)	87.0 (81.5 - 93.0)	79.5 (74.0 - 86.0)	$< 10^{-307}$
Waist-hip ratio	0.90 (0.86 - 0.93)	0.84 (0.79 - 0.89)	$< 10^{-307}$
Body mass index (kg/m^2)	24.9 (22.9 - 27.2)	22.9 (20.9 - 25.4)	$< 10^{-307}$
Blood Pressure			
Systolic BP† (mmHg)	121.0 (111.5 - 131.3)	111.0 (102.0 - 123.0)	$< 10^{-307}$
Diastolic BP† (mmHg)	76.0 (70.0 - 83.0)	69.0 (62.5 - 76.0)	$< 10^{-307}$
Mean BP† (mmHg)	91.0 (84.3 - 98.7)	83.0 (76.3 - 91.2)	$< 10^{-307}$
Lipid profiles			
Total cholesterol (mg/dL)	190.0 (168.0 - 213.0)	194.0 (172.0 - 218.0)	6.31×10^{-75}
HDL-cholesterol (mg/dL)	47.0 (40.0 - 54.0)	57.0 (49.0 - 66.0)	$< 10^{-307}$
LDL-cholesterol (mg/dL)	121.0 (101.0 - 142.0)	118.0 (98.0 - 140.0)	3.81×10^{-26}
Triglyceride (mg/dL)	107.0 (74.0 - 156.0)	83.0 (59.0 - 119.0)	$< 10^{-307}$
Glucose metabolism			
Fasting plasma glucose†† (mg/dL)	94.0 (89.0 - 99.0)	90.0 (86.0 - 95.0)	$< 10^{-307}$
HbA1C†† (%)	5.6 (5.4 - 5.9)	5.6 (5.4 - 5.8)	2.86×10^{-70}
Uric acid			
Uric acid††† (mg/dL)	6.3 (5.5 - 7.1)	4.7 (4.1 - 5.5)	$< 10^{-307}$
Renal function			
Creatinine (mg/dL)	0.88 (0.79 - 0.97)	0.60 (0.53 - 0.67)	$< 10^{-307}$
eGFR (mL/min/1.73 m^2)	93.2 (82.0 - 105.4)	106.6 (92.9 - 122.6)	$< 10^{-307}$
Albuminuria (mg/L)	8.3 (5.2 - 14.4)	8.9 (5.4 - 15.2)	3.10×10^{-15}
Liver function			
AST (U/L)	24.0 (21.0 - 29.0)	22.0 (19.0 - 26.0)	$< 10^{-307}$
ALT (U/L)	23.0 (17.0 - 33.0)	16.0 (12.0 - 22.0)	$< 10^{-307}$
γGT (U/L)	22.0 (16.0 - 34.0)	14.0 (11.0 - 21.0)	$< 10^{-307}$
Serum albumin (g/dL)	4.6 (4.4 - 4.7)	4.5 (4.3 - 4.6)	$< 10^{-307}$
Total bilirubin (mg/dL)	0.7 (0.6 - 0.9)	0.6 (0.5 - 0.7)	$< 10^{-307}$
Hematological parameters			
Leukocyte count (10^3/μL)	5.9 (4.9 - 6.9)	5.6 (4.6 - 6.6)	9.79×10^{-147}
Hematocrit (%)	44.9 (42.9 - 47.1)	40.0 (37.9 - 42.0)	$< 10^{-307}$
Platelet count (10^3/μL)	221.0 (190.0 - 256.0)	246.0 (211.0 - 287.0)	$< 10^{-307}$
Red blood cell count (10^6/μL)	5.1 (4.8 - 5.3)	4.5 (4.3 - 4.8)	$< 10^{-307}$
Hemoglobin (g/dL)	15.1 (14.4 - 15.8)	13.1 (12.4 - 13.8)	$< 10^{-307}$
Atherosclerotic risk factors			
Diabetes mellitus (%)	10.4%	6.7%	1.52×10^{-71}
Hypertension (%)	28.4%	15.8%	$< 10^{-307}$
Current smoking (%)	20.4%	2.8%	$< 10^{-307}$
Gout (%)	8.6%	0.4%	$< 10^{-307}$
Microalbuminuria (%)	11.3%	10.4%	7.20×10^{-5}
Metabolic syndrome (%)	24.5%	17.7%	2.24×10^{-107}

p value for continuous variables was calculated using Mann-Whitney U test and for category variables was calculated using Chi-Square Tests.

Participants were analyzed after the exclusion of those with a history of †hypertension, ††diabetes mellitus, and †††gout.

Data are presented as median (interquartile range).Abbreviations: BP, blood pressure; HDL, high-density lipoprotein; LDL, low-density lipoprotein; HbA1C, hemoglobin A1C; eGFR, estimated glomerular filtration rate; BUN, blood urea nitrogen; AST, aspartate aminotransferase; ALT, alanine aminotransferase; γGT, γ-Glutamyl transferase; BMI, body mass index.

Supplementary Table S2. Genome-wide significant association between *PCSK9* gene variants (minor allele frequency >0.01) on chromosome 1p32 and LDL-C level.

Chr	SNV	BP	Ref	BETA	SE	*P* value
1	rs7523141	55033125	C	-0.0047	0.0006	3.87×10^{-14}
1	rs7523242	55033276	C	-0.0047	0.0006	4.38×10^{-14}
1	rs7525649	55033483	C	-0.0047	0.0006	3.67×10^{-14}
1	rs10788994	55035303	C	-0.0047	0.0006	2.71×10^{-14}
1	rs2149041	55036464	C	-0.0046	0.0006	9.79×10^{-14}
1	rs4275490	55044266	C	-0.0066	0.0012	4.95×10^{-8}
1	rs58667756	55044601	A	-0.0066	0.0012	4.88×10^{-8}
1	rs557211	55048542	G	-0.0052	0.0007	3.75×10^{-12}
1	rs7543163	55049808	C	-0.0052	0.0008	1.29×10^{-11}
1	rs565436	55058928	G	-0.0067	0.0010	4.74×10^{-12}
1	rs41297885	55061167	G	-0.0084	0.0015	3.70×10^{-8}
1	rs597387	55065463	T	-0.0095	0.0016	1.51×10^{-9}

Abbreviations: Chr: chromosome, SNV: single nucleotide variation, bp: base pairs, Ref: reference allele, SE, standard error

P value adjusted for age, sex, body mass index and current smoking

Supplementary Table S3. Rare *PCSK9* gene variants selected for Mendelian randomization studies

Chr	SNV	End	Ref/Alt	Func.refGene	Gene.refGene	HWE	MAF	MM	Mm	mm	*P*	beta	SE	*P* value*
1	rs151193009	55509585	C/T	nonsynonymous SNV	exon2:c.C277T:p.R93C	0.4419	0.0027	120.98 ± 30.984 (73449)	106.76 ± 28.532 (405)	--	1.76×10^{-18}	-0.0579	0.0057	1.19×10^{-24}
1	rs768846693	55518412	C/A	nonsynonymous SNV	exon5:c.C747A:p.S249R	0.9078	0.0004	120.88 ± 31.021 (75324)	91.73 ± 28.176 (63)	--	1.18×10^{-9}	-0.1301	0.0143	1.12×10^{-19}
1	rs757143429	55523828	C/T	nonsynonymous SNV	exon8:c.C1300T:p.R434W	0.7454	0.0011	120.89 ± 31.023 (75239)	107.81 ± 30.246 (175)	--	1.47×10^{-7}	-0.0509	0.0086	3.46×10^{-9}
1	rs562556	55524237	G/A	nonsynonymous SNV	exon9:c.G1420A:p.V474I	0.9877	0.0035	120.85 ± 31.025 (74859)	121.04 ± 31.593 (519)	122.00 ± 0 (1)	0.9867	0.0015	0.0050	0.7653
1	rs72646525	55527093	C/T	nonsynonymous SNV	exon11:c.C1727T:p.P576L	4.98×10^{-13}	0.0009	120.83 ± 31.018 (74879)	127.47 ± 33.479 (133)	127.00 ± 11.314 (2)	0.0830	0.0220	0.0096	0.0217
1	rs765583923	55527099	T/A	nonsynonymous SNV	exon11:c.T1733A:p.V578E	2.57×10^{-45}	0.0005	120.87 ± 31.032 (75335)	109.13 ± 24.964 (68)	--	0.0014	-0.0383	0.0138	0.0055
1	rs367606156	55527158	G/A	nonsynonymous SNV	exon11:c.G1792A:p.A598T	0.5954	0.0019	120.89 ± 31.026 (74960)	115.22 ± 31.347 (281)	--	0.0023	-0.0200	0.0068	0.0033
1	rs201280059	55529132	A/G	nonsynonymous SNV	exon12:c.A1954G:p.N652D	0.8398	0.0007	120.85 ± 31.025 (74985)	119.83 ± 32.475 (105)	--	0.6290	-0.0028	0.0111	0.8001

Abbreviations: BP: base position, Ref/Alt: reference/alternate allele, HWE: Hardy-Weinberg equilibrium, MAF: minor allele frequency, other abbreviations as in Supplementary Table S1.
P: unadjusted*, P*:* adjusted for age, sex, BMI, and current smoking.
Data are presented as mean ± SD (number).

Supplementary Table S4. Association between low-density lipoprotein cholesterol (LDL-C) levels and diabetes mellitus (DM) status

	Unit	DM negative	DM positive	OR	95%, CI	beta	SE	*P** value
LDL-C levels	mg/dL	120.97 ± 30.66 (69391)	119.56 ± 34.92 (6046)	0.9936	(0.9927 - 0.9945)	-1.9993	0.1185	6.76×10^{-64}
	mmol/L	3.13 ± 0.79 (69391)	3.09 ± 0.9 (6046)	0.7810	(0.7538 - 0.8091)	-1.9993	0.1185	6.76×10^{-64}

OR: Odds ratio, CI: Confidence interval
P value: unadjusted, *P** value: adjusted for age, sex, BMI, and current smoking.
Data are presented as mean ± SD (number).

Supplementary Figure S1. Manhattan plots of genome-wide association study for LDL-C levels

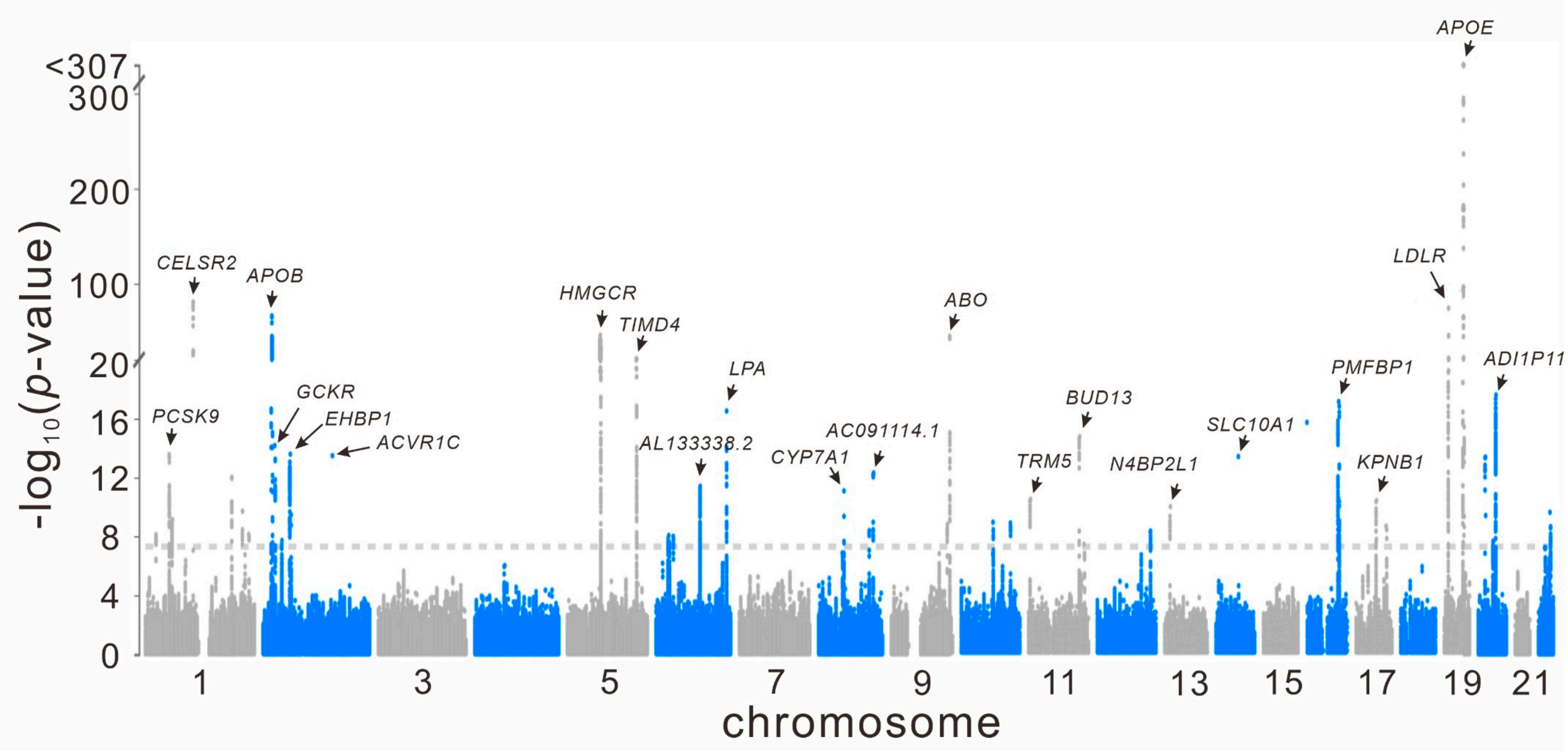

Supplementary Table S5. LDL-C levels and DM according to LDL-C levels -determining genotypes with genome-wide significance ($P < 5 \times 10^{-8}$) in Taiwan Biobank participants

							LDL-C levels			DM			DM adjusted LDL-C levels		
Chr	Position	Lead SNV	Ref/Alt	HWE	MAF	Candidate gene	BETA	SE	*P* value	BETA	SE	*P* value	BETA	SE	*P* value*
19	44,908,822	rs7412	C/T	0.2991	0.0737	*APOE*	-0.0912	0.0011	$< 10^{-307}$	-0.0717	0.0385	0.0627	-0.2596	0.0398	6.80×10^{-11}
1	109,274,968	rs12740374	G/T	0.2560	0.0665	*CELSR2*	-0.0222	0.0012	4.61×10^{-81}	0.0271	0.0394	0.4923	-0.0159	0.0397	0.6878
19	11,131,631	rs2738464	C/G	0.5968	0.2743	*LDLR, SPC24*	-0.0120	0.0007	3.76×10^{-75}	0.0573	0.0221	0.0094	0.0347	0.0222	0.1175
2	21,029,662	rs13306194	G/A	0.1738	0.1432	*APOB*	-0.0145	0.0008	5.47×10^{-67}	0.0574	0.0281	0.0409	0.0307	0.0282	0.2766
5	75,343,719	rs3064191	C/TTGTA	0.3309	0.4922	*HMGCR*	-0.0084	0.0006	2.31×10^{-46}	0.0124	0.0199	0.5325	-0.0022	0.0200	0.9125
9	133,266,456	rs2519093	C/T	0.6798	0.1822	*ABO*	0.0107	0.0008	2.89×10^{-45}	0.0081	0.0256	0.7514	0.0256	0.0258	0.3198
5	156,970,662	rs6882345	A/G	0.7144	0.2709	*TIMD4*	-0.0063	0.0007	1.26×10^{-21}	-0.0054	0.0224	0.8092	-0.0167	0.0225	0.4573
20	41,325,964	rs6124341	A/G	0.0877	0.2431	*ADI1P1*	-0.0059	0.0007	2.65×10^{-18}	0.0491	0.0229	0.0320	0.0397	0.0230	0.0842
16	72,194,607	rs3852789	A/C	0.6235	0.3159	*PMFBP1*	-0.0054	0.0006	8.89×10^{-18}	-0.0465	0.0215	0.0304	-0.0574	0.0216	0.0078
6	160,596,331	rs73596816	G/A	0.4500	0.0520	*LPA*	0.0111	0.0013	3.77×10^{-17}	0.0610	0.0437	0.1625	0.0817	0.0440	0.0632
16	377,772	rs375498857	C/A	0.0012	0.0154	*TMEM8A*	-0.0198	0.0024	1.49×10^{-16}	0.3642	0.0727	5.48E-07	0.3299	0.0731	6.36×10^{-6}
11	116,715,567	rs7350481	C/T	0.4572	0.2372	*BUD13*	0.0055	0.0007	1.56×10^{-15}	-0.0372	0.0237	0.1164	-0.0348	0.0238	0.1438
2	27,512,105	rs6547692	A/G	0.8163	0.4909	*GCKR*	0.0046	0.0006	6.52×10^{-15}	-0.0433	0.0199	0.0295	-0.0348	0.0200	0.0811
2	62,679,481	rs10184918	G/A	0.3777	0.3116	*EHBP1*	-0.0048	0.0006	2.46×10^{-14}	0.0010	0.0214	0.9613	-0.0082	0.0215	0.7026
1	55,035,303	rs10788994	T/C	0.5653	0.3471	*PCSK9*	-0.0047	0.0006	1.99×10^{-14}	0.0098	0.0209	0.6380	0.0014	0.0210	0.9476
2	157,625,480	rs10164853	A/G	0.6386	0.3154	*ACVR1C*	-0.0048	0.0006	3.26×10^{-14}	0.0111	0.0213	0.6011	0.0012	0.0214	0.9569
14	69,778,476	rs2296651	G/A	0.0044	0.0966	*SLC10A1*	-0.0075	0.0010	3.70×10^{-14}	-0.0859	0.0342	0.0121	-0.0994	0.0343	0.0038
20	17,565,577	rs6105777	T/G	0.3222	0.0789	*BFSP1, AL132765.2*	-0.0082	0.0011	4.07×10^{-14}	0.0058	0.0368	0.8756	-0.0131	0.0370	0.7229
8	125,467,120	rs6982502	T/C	0.4841	0.4366	*AC091114.1*	0.0043	0.0006	4.88×10^{-13}	-0.0259	0.0200	0.1959	-0.0173	0.0201	0.3892
1	196,729,914	rs10801558	T/G	0.5773	0.4437	*CFH*	-0.0042	0.0006	9.38×10^{-13}	-0.0107	0.0200	0.5916	-0.0193	0.0201	0.3350

6	100,970,961	rs629192	A/G	0.1220	0.2115	*AL133338.2*	0.0050	0.0007	3.84×10^{-12}	-0.0466	0.0245	0.0576	-0.0386	0.0246	0.1169
8	58,493,931	rs112784971	C/T	0.8346	0.2299	*CYP7A1*	-0.0048	0.0007	7.95×10^{-12}	0.0178	0.0235	0.4484	0.0077	0.0236	0.7440
16	71,419,076	rs184643955	G/A	0.4043	0.0175	*LINC02136*	0.0150	0.0023	2.60×10^{-11}	0.1164	0.0723	0.1077	0.1426	0.0727	0.0499
11	5,670,559	rs16934050	A/C	0.8918	0.4562	*AC104389.5, TRIM5*	-0.0039	0.0006	3.00×10^{-11}	-0.0629	0.0201	0.0018	-0.0707	0.0202	0.0005
17	47,685,707	rs4794048	A/C	0.6781	0.3458	*KPNB1*	0.0041	0.0006	3.65×10^{-11}	0.0074	0.0210	0.7226	0.0124	0.0211	0.5552
13	32,402,221	rs10492397	G/A	0.0366	0.3549	*N4BP2L1*	-0.0040	0.0006	9.82×10^{-11}	0.0102	0.0211	0.6269	0.0021	0.0212	0.9227
1	220,811,022	rs11118607	C/T	0.4088	0.1248	*MARC1, AL445423.3*	-0.0057	0.0009	2.03×10^{-10}	0.0072	0.0301	0.8106	-0.0027	0.0302	0.9288
22	42,138,592	rs2743450	A/G	0.2771	0.4507	*AC254562.2, AC254562.3*	-0.0038	0.0006	2.39×10^{-10}	0.0001	0.0201	0.9944	-0.0065	0.0202	0.7485
20	17,865,277	rs2328223	A/C	0.7142	0.1977	*AL035045.1*	0.0046	0.0007	3.97×10^{-10}	-0.0406	0.0250	0.1054	-0.0335	0.0252	0.1837
1	62,586,017	rs12030293	C/T	0.1299	0.2005	*DOCK7*	-0.0045	0.0007	7.61×10^{-10}	0.0484	0.0244	0.0474	0.0442	0.0245	0.0715
10	72,879,221	rs11000435	C/T	0.2708	0.2124	*MCU*	-0.0044	0.0007	1.12×10^{-9}	0.0069	0.0244	0.7758	-0.0021	0.0245	0.9318
10	112,153,464	rs2297991	C/T	0.9860	0.2844	*GPAM*	0.0039	0.0006	1.28×10^{-9}	0.0109	0.0220	0.6207	0.0191	0.0221	0.3873
9	127,966,535	rs74987020	CAG/C	0.2797	0.2188	*FAM102A*	0.0043	0.0007	1.57×10^{-9}	-0.0111	0.0241	0.6457	-0.0036	0.0242	0.8816
17	69,086,125	rs12162136	A/G	0.9118	0.4261	*ABCA6*	0.0036	0.0006	2.06×10^{-9}	-0.0284	0.0202	0.1604	-0.0224	0.0203	0.2712
8	115,658,120	rs3808477	C/T	0.0884	0.3071	*TRPS1*	-0.0037	0.0006	4.21×10^{-9}	-0.0295	0.0217	0.1736	-0.0356	0.0218	0.1020
12	120,977,112	rs2255531	G/A	0.1431	0.3955	*HNF1A-AS1*	0.0035	0.0006	5.14×10^{-9}	-0.0246	0.0205	0.2306	-0.0172	0.0206	0.4046
1	234,712,862	rs486142	A/G	0.5409	0.2251	*AL160408.6*	-0.0041	0.0007	7.54×10^{-9}	-0.0095	0.0238	0.6908	-0.0162	0.0239	0.4987
1	25,474,918	rs61775184	T/C	0.0233	0.2674	*MACO1*	0.0039	0.0007	7.85×10^{-9}	0.0618	0.0225	0.0061	0.0681	0.0226	0.0026
19	45,970,068	rs149521600	T/C	0.9893	0.0100	*NOVA2*	-0.0172	0.0030	8.68×10^{-9}	0.0910	0.1000	0.3627	0.0615	0.1005	0.5406
6	29,969,017	rs41267238	T/TC	0.4499	0.3046	*MICD*	-0.0037	0.0006	8.87×10^{-9}	0.0075	0.0215	0.7279	-0.0011	0.0216	0.9594
6	39,942,978	rs139042980	C/T	0.6385	0.0167	*TUBBP9*	-0.0130	0.0023	1.01×10^{-8}	0.0049	0.0759	0.9485	-0.0194	0.0762	0.7992
17	47,294,237	rs999323	C/T	0.8431	0.4651	*ITGB3, AC068234.1*	0.0033	0.0006	1.62×10^{-8}	0.0206	0.0200	0.3034	0.0254	0.0201	0.2052
2	43,725,556	rs11695642	G/T	0.5352	0.1146	*PLEKHH2*	0.0052	0.0009	1.97×10^{-8}	-0.0320	0.0317	0.3117	-0.0220	0.0318	0.4891
20	35,555,321	rs224419	A/G	0.1337	0.2228	*ERGIC3*	-0.0039	0.0007	2.14×10^{-8}	0.0214	0.0236	0.3654	0.0133	0.0237	0.5758
19	44,217,383	rs144882440	A/G	0.4438	0.0119	*ZNF227*	-0.0151	0.0027	2.62×10^{-8}	-0.0751	0.0942	0.4251	-0.1050	0.0948	0.2681

6	31,357,968	rs74618856	C/T	0.4175	0.4232	*AL671883.2*	-0.0033	0.0006	2.78×10^{-8}	-0.0078	0.0201	0.6968	-0.0149	0.0202	0.4612
11	126,371,957	rs72085277	TTCTG/T	0.7274	0.3577	*ST3GAL4*	0.0034	0.0006	3.45×10^{-8}	-0.0122	0.0208	0.5586	-0.0063	0.0209	0.7617

P value: adjusted for age, sex, BMI, and current smoking
P value*: adjusted for age, sex, BMI, current smoking, and LDL-C levels

Supplementary Table S6. Association of the *PCSK9* rs10788994 genotype with metabolic and hematological phenotypes

Clinical and laboratory parameters	beta	SE	*P* value
Anthropology			
Age (years)	0.1026	0.0580	0.0767
Waist circumference (cm)	-0.0014	0.0276	0.9588
Waist-hip ratio	0.0001	0.0003	0.7223
Body mass index (kg/m^2)	-0.0297	0.0196	0.1297
Blood Pressure			
Systolic BP[†] (mmHg)	-0.0793	0.0823	0.3354
Diastolic BP[†] (mmHg)	-0.0709	0.0538	0.1876
Mean BP[†] (mmHg)	-0.0737	0.0591	0.2124
Lipid profiles			
Total cholesterol (mg/dL)	-0.0028	0.0004	9.25×10^{-12}
HDL-cholesterol (mg/dL)	0.0010	0.0005	0.0513
LDL-cholesterol (mg/dL)	-0.0047	0.0006	1.99×10^{-14}
Triglyceride (mg/dL)	-0.0021	0.0012	0.0708
Glucose metabolism			
Fasting plasma glucose[††] (mg/dL)	0.011	0.0811	0.8921
HbA1C[††] (%)	0.0003	0.0032	0.9235
Uric acid			
Uric acid[†††] (mg/dL)	0.0014	0.0060	0.8114
Renal function			
Creatinine (mg/dL)	0.0017	0.0013	0.2187
eGFR (mL/min/1.73 m^2)	-0.3098	0.1174	0.0083
Albuminuria (mg/L)	0.0027	0.0024	0.2615
Liver function			
AST (U/L)	-0.131	0.0651	0.0442
ALT (U/L)	-0.2161	0.101	0.0324
γGT (U/L)	0.1301	0.1531	0.3954
Serum albumin (g/dL)	4.30×10^{-5}	0.0012	0.9718
Total bilirubin (mg/dL)	0.0002	0.0015	0.8875
Hematological parameters			
Leukocyte count (10^3/μL)	-0.008	0.0084	0.3394
Hematocrit (%)	-0.0082	0.0190	0.6668
Platelet count (10^3/μL)	-0.0316	0.3110	0.9190
Red blood cell count (10^6/μL)	4.50×10^{-5}	0.0024	0.9852
Hemoglobin (g/dL)	0.0082	0.0067	0.2238

P value: adjusted for age, sex, BMI, and current smoking; Age: adjusted for BMI and current smoking; and BMI: adjusted for age and smoking. Participants were analyzed after the exclusion of those with a history of [†]hypertension, [††]diabetes mellitus, and [†††]gout. Abbreviations: SE, standard error; BP, blood pressure; HDL, high-density lipoprotein; LDL, low-density lipoprotein; HbA1C, hemoglobin A1C; eGFR, estimated glomerular filtration rate; BUN, blood urea nitrogen; AST, aspartate aminotransferase; ALT, alanine aminotransferase; γGT, γ-Glutamyl transferase; BMI, body mass index.

Supplementary Table S7. Association between *PCSK9* rs10788994 genotype and lifestyle and atherosclerotic risk factors

rs10788994	TT	TC	CC	*P1* value	beta	SE	*P2* value
Diabetes mellitus (%)	7.90%	8.10%	8.00%	0.7176	0.0098	0.0209	0.6380
Hypertension (%)	20.30%	20.20%	20.60%	0.6855	-0.0021	0.0149	0.8902
Current smoking (%)	9.00%	9.00%	9.30%	0.7566	0.0107	0.0199	0.5914
Gout (%)	3.30%	3.40%	3.30%	0.6419	0.0188	0.0313	0.5487
Microalbuminuria (%)	10.60%	10.70%	11.20%	0.3203	0.0242	0.0178	0.1736
Metabolic syndrome (%)	20.30%	20.10%	19.70%	0.3803	-0.0210	0.0155	0.1765

P1 value: unadjusted.

P2 value adjusted for age, sex, body mass index and current smoking. Current smoking: adjusted for age, BMI and sex

Supplementary Table S8. Association of the *PCSK9* rs151193009 genotype with metabolic and hematological phenotypes

Clinical and laboratory parameters	beta	SE	*P* value
Anthropology			
Age (years)	1.3056	0.5311	0.014
Waist circumference (cm)	0.1500	0.2526	0.5527
Waist-hip ratio	0.0005	0.0026	0.8590
Body mass index (kg/m^2)	0.0624	0.1795	0.7280
Blood Pressure			
Systolic BP† (mmHg)	0.6235	0.7652	0.4152
Diastolic BP† (mmHg)	0.5638	0.4996	0.2591
Mean BP† (mmHg)	0.5837	0.5490	0.2877
Lipid profiles			
Total cholesterol (mg/dL)	-0.0367	0.0038	2.30×10^{-22}
HDL-cholesterol (mg/dL)	0.0008	0.0045	0.8604
LDL-cholesterol (mg/dL)	-0.0579	0.0057	1.19×10^{-24}
Triglyceride (mg/dL)	-0.0057	0.0106	0.5899
Glucose metabolism			
Fasting plasma glucose†† (mg/dL)	0.0870	0.7410	0.9066
HbA1C†† (%)	-0.0112	0.0297	0.7055
Uric acid			
Uric acid††† (mg/dL)	0.0292	0.0546	0.5934
Renal function			
Creatinine (mg/dL)	0.0172	0.0122	0.1576
eGFR (mL/min/1.73 m^2)	-1.0881	1.0752	0.3116
Albuminuria (mg/L)	0.0136	0.0223	0.5400
Liver function			
AST (U/L)	0.5948	0.597	0.3191
ALT (U/L)	0.9244	0.9236	0.3169
γGT (U/L)	0.1557	1.4115	0.9122
Serum albumin (g/dL)	-0.0062	0.0111	0.5757
Total bilirubin (mg/dL)	-0.0087	0.0134	0.5134
Hematological parameters			
Leukocyte count (10^3/μL)	-0.0198	0.0765	0.7955
Hematocrit (%)	0.0208	0.1739	0.9048
Platelet count (10^3/μL)	-4.5149	2.8500	0.1132
Red blood cell count (10^6/μL)	0.0161	0.0219	0.4645
Hemoglobin (g/dL)	-0.0081	0.0616	0.8955

Abbreviations, adjustment and participant recruitment as in Supplementary Table S6

Supplementary Table S9. Association between *PCSK9* rs151193009 genotype and lifestyle and atherosclerotic risk factors

rs151193009	CC	CT	*P1* value	beta	SE	*P2* value
Diabetes mellitus (%)	8.00%	9.60%	0.2258	0.1664	0.1751	0.3422
Hypertension (%)	20.30%	24.40%	0.0371	0.1755	0.1269	0.1665
Current smoking (%)	9.10%	8.40%	0.6463	-0.0690	0.1881	0.7139
Gout (%)	3.30%	3.00%	0.6844	-0.1888	0.3018	0.5316
Microalbuminuria (%)	10.70%	10.40%	0.8295	-0.0514	0.1648	0.7551
Metabolic syndrome (%)	20.10%	23.00%	0.1529	0.1419	0.1320	0.2827

Abbreviations and adjustment as in Supplementary Table S7

Supplementary Table S10. Association of the *PCSK9* rs557211 genotype with metabolic and hematological phenotypes

Clinical and laboratory parameters	beta	SE	*P* value
Anthropology			
Age (years)	0.1064	0.0699	0.1282
Waist circumference (cm)	-0.0019	0.0333	0.9534
Waist-hip ratio	0.0003	0.0003	0.4650
Body mass index (kg/m^2)	0.0017	0.0237	0.9442
Blood Pressure			
Systolic BP† (mmHg)	-0.0258	0.0990	0.7946
Diastolic BP† (mmHg)	0.0015	0.0647	0.9817
Mean BP† (mmHg)	-0.0076	0.0711	0.9148
Lipid profiles			
Total cholesterol (mg/dL)	-0.0034	0.0005	1.32×10^{-11}
HDL-cholesterol (mg/dL)	0.0006	0.0006	0.3348
LDL-cholesterol (mg/dL)	-0.0052	0.0007	3.88×10^{-12}
Triglyceride (mg/dL)	-0.0008	0.0014	0.5435
Glucose metabolism			
Fasting plasma glucose†† (mg/dL)	-0.0100	0.0977	0.9185
HbA1C†† (%)	-0.0015	0.0039	0.7017
Uric acid			
Uric acid††† (mg/dL)	0.0055	0.0072	0.4433
Renal function			
Creatinine (mg/dL)	-0.0008	0.0016	0.6138
eGFR (mL/min/1.73 m^2)	-0.1452	0.1418	0.3056
Albuminuria (mg/L)	-0.0021	0.0029	0.4742
Liver function			
AST (U/L)	-0.0504	0.0784	0.5203
ALT (U/L)	0.0132	0.1216	0.9135
γGT (U/L)	0.0387	0.1850	0.8344
Serum albumin (g/dL)	-0.0012	0.0015	0.4298
Total bilirubin (mg/dL)	-0.0008	0.0018	0.6473
Hematological parameters			
Leukocyte count (10^3/μL)	0.0019	0.0101	0.8483
Hematocrit (%)	-0.0180	0.0229	0.4327
Platelet count (10^3/μL)	-0.0052	0.3751	0.9890
Red blood cell count (10^6/μL)	0.0018	0.0029	0.5411
Hemoglobin (g/dL)	0.0058	0.0081	0.4725

Abbreviations, adjustment and participant recruitment as in Supplementary Table S6.

Supplementary Table S11. Association between *PCSK9* rs557211 genotype and lifestyle and atherosclerotic risk factors

rs557211	TT	TG	GG	*P1* value	beta	SE	*P2* value
Diabetes mellitus (%)	7.98%	8.05%	8.36%	0.7597	0.0063	0.0252	0.8028
Hypertension (%)	20.39%	20.10%	18.91%	0.1365	-0.0439	0.0181	0.0153
Current smoking (%)	9.03%	9.09%	9.34%	0.8504	0.0178	0.0241	0.4589
Gout (%)	3.35%	3.25%	3.47%	0.7213	-0.0128	0.0381	0.7367
Microalbuminuria (%)	10.68%	10.66%	11.29%	0.5958	0.0091	0.0215	0.6701
Metabolic syndrome (%)	20.07%	20.30%	19.72%	0.6656	0.0006	0.0187	0.9735

Abbreviations and adjustment as in Supplementary Table S7

Supplementary Table S12. Association of the *PCSK9* rs768846693 genotype with metabolic and hematological phenotypes

Clinical and laboratory parameters	beta	SE	*P* value
Anthropology			
Age (years)	1.8598	1.3437	0.1663
Waist circumference (cm)	0.1836	0.6393	0.7740
Waist-hip ratio	0.0027	0.0067	0.6896
Body mass index (kg/m^2)	0.3193	0.4543	0.4822
Blood Pressure			
Systolic BP† (mmHg)	-0.8296	1.9330	0.6678
Diastolic BP† (mmHg)	-1.4447	1.2628	0.2526
Mean BP† (mmHg)	-1.2397	1.3874	0.3716
Lipid profiles			
Total cholesterol (mg/dL)	-0.0679	0.0096	1.21×10^{-12}
HDL-cholesterol (mg/dL)	0.0050	0.0115	0.6621
LDL-cholesterol (mg/dL)	-0.1301	0.0143	1.12×10^{-19}
Triglyceride (mg/dL)	0.0388	0.0268	0.1472
Glucose metabolism			
Fasting plasma glucose†† (mg/dL)	3.3826	1.9515	0.0830
HbA1C†† (%)	0.0775	0.0782	0.3217
Uric acid			
Uric acid††† (mg/dL)	-0.0460	0.1384	0.7394
Renal function			
Creatinine (mg/dL)	0.0245	0.0313	0.4331
eGFR (mL/min/1.73 m^2)	2.9587	2.7239	0.2774
Albuminuria (mg/L)	-0.0718	0.0563	0.2020
Liver function			
AST (U/L)	-1.7225	1.5094	0.2538
ALT (U/L)	-1.6459	2.3396	0.4817
γGT (U/L)	3.6903	3.5567	0.2995
Serum albumin (g/dL)	-0.0327	0.0280	0.2433
Total bilirubin (mg/dL)	-0.0131	0.0338	0.6981
Hematological parameters			
Leukocyte count (10^3/μL)	0.1836	0.1934	0.3425
Hematocrit (%)	-0.315	0.4401	0.4741
Platelet count (10^3/μL)	-4.1271	7.2033	0.5667
Red blood cell count (10^6/μL)	0.0712	0.0556	0.2004
Hemoglobin (g/dL)	-0.1363	0.1558	0.3818

Abbreviations, adjustment and participant recruitment as in Supplementary Table S6.

Supplementary Table S13. Association between *PCSK9* rs768846693 genotype and lifestyle and atherosclerotic risk factors

rs768846693	CC	CA	*P1* value	beta	SE	*P2* value
Diabetes mellitus (%)	8.00%	19.00%	0.0012	0.9596	0.3487	0.0059
Hypertension (%)	20.30%	22.20%	0.6991	-0.0383	0.338	0.9098
Current smoking (%)	9.10%	3.20%	0.1036	-1.0400	0.7348	0.1569
Gout (%)	3.30%	3.20%	0.9467	-0.0919	0.7474	0.9022
Microalbuminuria (%)	10.70%	7.90%	0.4773	-0.4027	0.4727	0.3943
Metabolic syndrome (%)	20.10%	31.70%	0.0214	0.6881	0.3130	0.0279

Abbreviations and adjustment as in Supplementary Table S7

Supplementary Table S14. Association of the *PCSK9* rs757143429 genotype with metabolic and hematological phenotypes

Clinical and laboratory parameters	beta	SE	*P* value
Anthropology			
Age (years)	0.0189	0.8068	0.9813
Waist circumference (cm)	-0.0130	0.3838	0.9730
Waist-hip ratio	-0.0022	0.0040	0.5789
Body mass index (kg/m^2)	-0.2996	0.2727	0.2720
Blood Pressure			
Systolic BP† (mmHg)	0.8674	1.1342	0.4444
Diastolic BP† (mmHg)	0.0035	0.7410	0.9963
Mean BP† (mmHg)	0.2914	0.8141	0.7204
Lipid profiles			
Total cholesterol (mg/dL)	-0.0308	0.0057	7.80×10^{-8}
HDL-cholesterol (mg/dL)	-0.0065	0.0069	0.3414
LDL-cholesterol (mg/dL)	-0.0509	0.0086	3.46×10^{-9}
Triglyceride (mg/dL)	0.0006	0.0161	0.9691
Glucose metabolism			
Fasting plasma glucose†† (mg/dL)	-0.6723	1.1346	0.5535
HbA1C†† (%)	-0.0256	0.0455	0.5737
Uric acid			
Uric acid††† (mg/dL)	-0.0780	0.0824	0.3442
Renal function			
Creatinine (mg/dL)	-0.0222	0.0188	0.2368
eGFR (mL/min/1.73 m^2)	1.65950	1.6353	0.3102
Albuminuria (mg/L)	-6.7759	12.825	0.5973
Liver function			
AST (U/L)	1.9382	0.906	0.0324
ALT (U/L)	1.8749	1.4084	0.1831
γGT (U/L)	3.0228	2.1340	0.1566
Serum albumin (g/dL)	-0.0043	0.0168	0.7974
Total bilirubin (mg/dL)	0.0035	0.0203	0.8616
Hematological parameters			
Leukocyte count (10^3/μL)	0.0964	0.1161	0.4066
Hematocrit (%)	-0.3645	0.2642	0.1677
Platelet count (10^3/μL)	1.8928	4.3248	0.6616
Red blood cell count (10^6/μL)	-0.0332	0.0334	0.3205
Hemoglobin (g/dL)	-0.1275	0.0935	0.1728

Abbreviations, adjustment and participant recruitment as in Supplementary Table S6

Supplementary Table S15. PCSK9 Association between *PCSK9* rs757143429 genotype and lifestyle and atherosclerotic risk factors

rs757143429	CC	CT	*P1* value	beta	SE	*P2* value
Diabetes mellitus (%)	8.00%	8.60%	0.7854	0.1533	0.2838	0.5890
Hypertension (%)	20.30%	16.60%	0.2239	-0.2127	0.2219	0.3377
Current smoking (%)	9.10%	9.70%	0.7634	0.0996	0.2704	0.7126
Gout (%)	3.30%	1.70%	0.2337	-0.6481	0.5925	0.2740
Microalbuminuria (%)	10.70%	8.00%	0.2565	-0.2870	0.2817	0.3084
Metabolic syndrome (%)	20.10%	21.10%	0.7359	0.1978	0.2115	0.3497

Abbreviations and adjustment as in Supplementary Table S7

Supplementary Table S16. Association of the *PCSK9* rs565436 genotype with metabolic and hematological phenotypes

Clinical and laboratory parameters	beta	SE	*P* value
Anthropology			
Age (years)	0.1103	0.0906	0.2236
Waist circumference (cm)	0.0462	0.0432	0.2844
Waist-hip ratio	0.0004	0.0005	0.4319
Body mass index (kg/m^2)	0.0261	0.0307	0.3947
Blood Pressure			
Systolic BP† (mmHg)	0.2628	0.1285	0.0409
Diastolic BP† (mmHg)	0.1016	0.0839	0.2263
Mean BP† (mmHg)	0.1553	0.0922	0.0922
Lipid profiles			
Total cholesterol (mg/dL)	-0.0043	0.0006	1.69×10^{-11}
HDL-cholesterol (mg/dL)	0.0004	0.0008	0.5950
LDL-cholesterol (mg/dL)	-0.0067	0.0010	3.81×10^{-12}
Triglyceride (mg/dL)	-0.0019	0.0018	0.2893
Glucose metabolism			
Fasting plasma glucose†† (mg/dL)	0.1982	0.1261	0.1159
HbA1C†† (%)	0.0025	0.0051	0.6191
Uric acid			
Uric acid††† (mg/dL)	0.0036	0.0093	0.7014
Renal function			
Creatinine (mg/dL)	0.0002	0.0021	0.9396
eGFR (mL/min/1.73 m^2)	-0.1853	0.1838	0.3132
Albuminuria (mg/L)	-0.0019	0.0038	0.6172
Liver function			
AST (U/L)	-0.1743	0.1015	0.0861
ALT (U/L)	-0.2576	0.1575	0.1019
γGT (U/L)	0.1478	0.2388	0.5360
Serum albumin (g/dL)	-0.0009	0.0019	0.6513
Total bilirubin (mg/dL)	0.0017	0.0023	0.4656
Hematological parameters			
Leukocyte count (10^3/μL)	0.0095	0.0130	0.4681
Hematocrit (%)	-0.0257	0.0297	0.387
Platelet count (10^3/μL)	0.5582	0.4849	0.2497
Red blood cell count (10^6/μL)	-0.0036	0.0038	0.3415
Hemoglobin (g/dL)	-0.0074	0.0105	0.4835

Abbreviations, adjustment and participant recruitment as in Supplementary Table S6

Supplementary Table S17. PCSK9 Association between *PCSK9* rs565436 genotype and lifestyle and atherosclerotic risk factors

rs565436	AA	AG	GG	*P1* value	beta	SE	*P2* value
Diabetes mellitus (%)	8.00%	7.90%	9.20%	0.3920	-0.0103	0.0328	0.7531
Hypertension (%)	20.20%	20.80%	19.60%	0.1642	0.0262	0.0232	0.2590
Current smoking (%)	9.00%	9.00%	10.10%	0.5648	0.0155	0.0312	0.6186
Gout (%)	3.30%	3.60%	2.20%	0.0475	0.0373	0.0487	0.4438
Microalbuminuria (%)	10.70%	10.80%	11.40%	0.7936	0.0098	0.0278	0.7252
Metabolic syndrome (%)	20.00%	20.50%	21.80%	0.1818	0.0377	0.0240	0.1172

Abbreviations and adjustment as in Supplementary Table S7

Supplementary Table S18. Association of the *PCSK9* rs505151 genotype with metabolic and hematological phenotypes

Clinical and laboratory parameters	beta	SE	*P* value
Anthropology			
Age (years)	-0.1558	0.1222	0.2025
Waist circumference (cm)	-0.0208	0.0582	0.7209
Waist-hip ratio	0.0002	0.0006	0.7554
Body mass index (kg/m^2)	0.0123	0.0413	0.7659
Blood Pressure			
Systolic BP† (mmHg)	-0.1535	0.1738	0.3771
Diastolic BP† (mmHg)	-0.1778	0.1135	0.1172
Mean BP† (mmHg)	-0.1697	0.1247	0.1736
Lipid profiles			
Total cholesterol (mg/dL)	0.0035	0.0009	6.60×10^{-5}
HDL-cholesterol (mg/dL)	-0.0013	0.0010	0.2271
LDL-cholesterol (mg/dL)	0.0065	0.0013	5.89×10^{-7}
Triglyceride (mg/dL)	0.0007	0.0024	0.7605
Glucose metabolism			
Fasting plasma glucose†† (mg/dL)	-0.0878	0.1707	0.6069
HbA1C†† (%)	-0.0060	0.0068	0.3785
Uric acid			
Uric acid††† (mg/dL)	0.0149	0.0126	0.2363
Renal function			
Creatinine (mg/dL)	0.0007	0.0028	0.8191
eGFR (mL/min/1.73 m^2)	0.2694	0.2478	0.2769
Albuminuria (mg/L)	0.0003	0.0051	0.9575
Liver function			
AST (U/L)	-0.2159	0.1369	0.1148
ALT (U/L)	-0.4063	0.2124	0.0558
γGT (U/L)	0.0236	0.3232	0.9419
Serum albumin (g/dL)	0.0032	0.0026	0.2137
Total bilirubin (mg/dL)	0.0030	0.0031	0.3378
Hematological parameters			
Leukocyte count (10^3/μL)	0.0448	0.0176	0.0109
Hematocrit (%)	0.0309	0.0400	0.4409
Platelet count (10^3/μL)	0.9029	0.6545	0.1677
Red blood cell count (10^6/μL)	-0.0057	0.0051	0.2625
Hemoglobin (g/dL)	0.0166	0.0142	0.2427

Abbreviations, adjustment and participant recruitment as in Supplementary Table S6

Supplementary Table S19. Association between *PCSK9* rs505151 genotype and lifestyle and atherosclerotic risk factors

rs505151	AA	AG	GG	*P1* value	beta	SE	*P2* value
Diabetes mellitus (%)	8.08%	7.38%	12.09%	0.0092	-0.0574	0.045	0.2021
Hypertension (%)	20.29%	20.03%	21.40%	0.7928	-0.0051	0.0316	0.8727
Current smoking (%)	9.02%	9.42%	8.37%	0.4830	0.0364	0.0417	0.3828
Gout (%)	3.37%	2.95%	2.79%	0.1362	-0.1434	0.0700	0.0404
Microalbuminuria (%)	10.67%	10.98%	9.77%	0.6420	0.0250	0.0373	0.5021
Metabolic syndrome (%)	20.18%	19.53%	23.72%	0.1723	-0.0251	0.0329	0.4454

Abbreviations and adjustment as in Supplementary Table S7

Supplementary Table S20. F-statistics for *PCSK9* variants

Chr	Gene	SNP	position	Allele	MAF	HWE	beta	SE	*P* value*	F-statistic	R^2
1	*PCSK9*	rs10788994	55500976	C/T	0.3475	0.7616	-0.0047	0.0006	1.99×10^{-14}	717.7074	0.0460
1	*PCSK9*	rs151193009	55509585	C/T	0.0027	0.4419	-0.0579	0.0057	1.19×10^{-24}	725.9939	0.0469
1	*PCSK9*	rs557211	55514215	T/G	0.1916	0.8433	-0.0052	0.0007	3.75×10^{-12}	720.4977	0.0458
1	*PCSK9*	rs768846693	55518412	C/A	0.0004	0.9078	-0.1301	0.0143	1.12×10^{-19}	729.4731	0.0462
1	*PCSK9*	rs757143429	55523828	C/T	0.0011	0.7454	-0.0509	0.0086	3.46×10^{-9}	719.6079	0.0455
1	*PCSK9*	rs565436	55524601	G/A	0.1033	0.3577	-0.0067	0.0010	3.81×10^{-12}	716.3462	0.0457
1	*PCSK9*	rs505151	55529187	G/A	0.0534	0.9202	0.0065	0.0013	5.89×10^{-7}	716.5329	0.0454

*LDL-C adjusted for sex, age, body mass index and current smoking

Supplementary Figure S2. Scatter plots for putative causal relationships between low density lipoprotein cholesterol (LDL-C) level-determining alleles on diabetic mellitus (DM) using (A) *PCSK9* variants and (B) LDL-C level GWAS-determined variants. We exhibit the effect sizes of LDL-C level-determining alleles on LDL-C level and DM. Lines represent one standard error (s.e.).

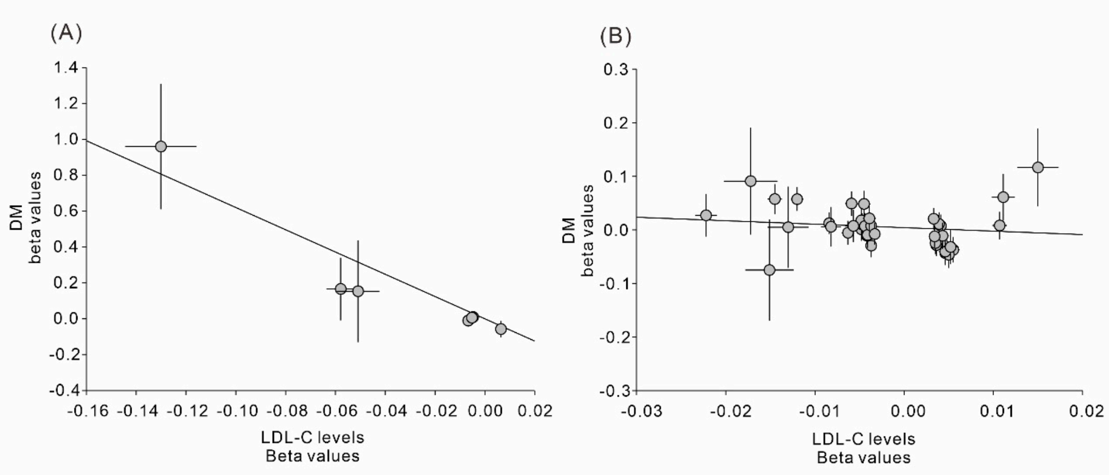

Supplementary Table S21. Cochran's Q tests for heterogeneity

Exposure[E]	Outcome[O]	Instrumental variables	Analysis Method*	Coefficient*	Standard error*	95% confident interval*	*P* value*	Cochran's Q	*P* value**
LDC-C	DM	WGRS_PCSK9_7SNPs	IVW method	-3.9309	1.5141	(-7.6357, -0.2261)	9.42×10^{-3}	4.0351	0.6719
			Egger regression, slope	-5.3116	1.0983	(-8.1349, -2.4883)	0.0047	10.7580	0.0961
		WGRS_PCSK9_41SNPs	IVW method	-2.2050	0.6149	(-3.4476, -0.9623)	3.36×10^{-4}	39.9988	0.4700
			Egger regression, slope	-2.3301	0.6374	(-3.6192, -1.0409)	0.0008	52.8469	0.0839

IVW: Inverse-variance weighted
*All with fixed effect
***P* value: *P* value for the Cochran's Q tests

Supplementary Figure S3. Funnel plots of the instrumental variable (IV) strength against IV estimates for each genetic variant separately for standard MR for the LDL-C level on DM. (A) *PCSK9* variants and (B) LDL-C level GWAS-determined variants. Horizontal lines represent 95% confident intervals for the IV estimates. Solid vertical lines are at the null, and dashed vertical lines are the (fixed-effect) inverse-variance weighted estimates. Instrumental variable strength: $(\frac{\widehat{\beta x}\ |G}{SE\ (\widehat{\beta Y}|G)})$ and instrumental variable estimates: $(\frac{\widehat{\beta Y}\ |G}{\widehat{\beta X}\ |G})$.

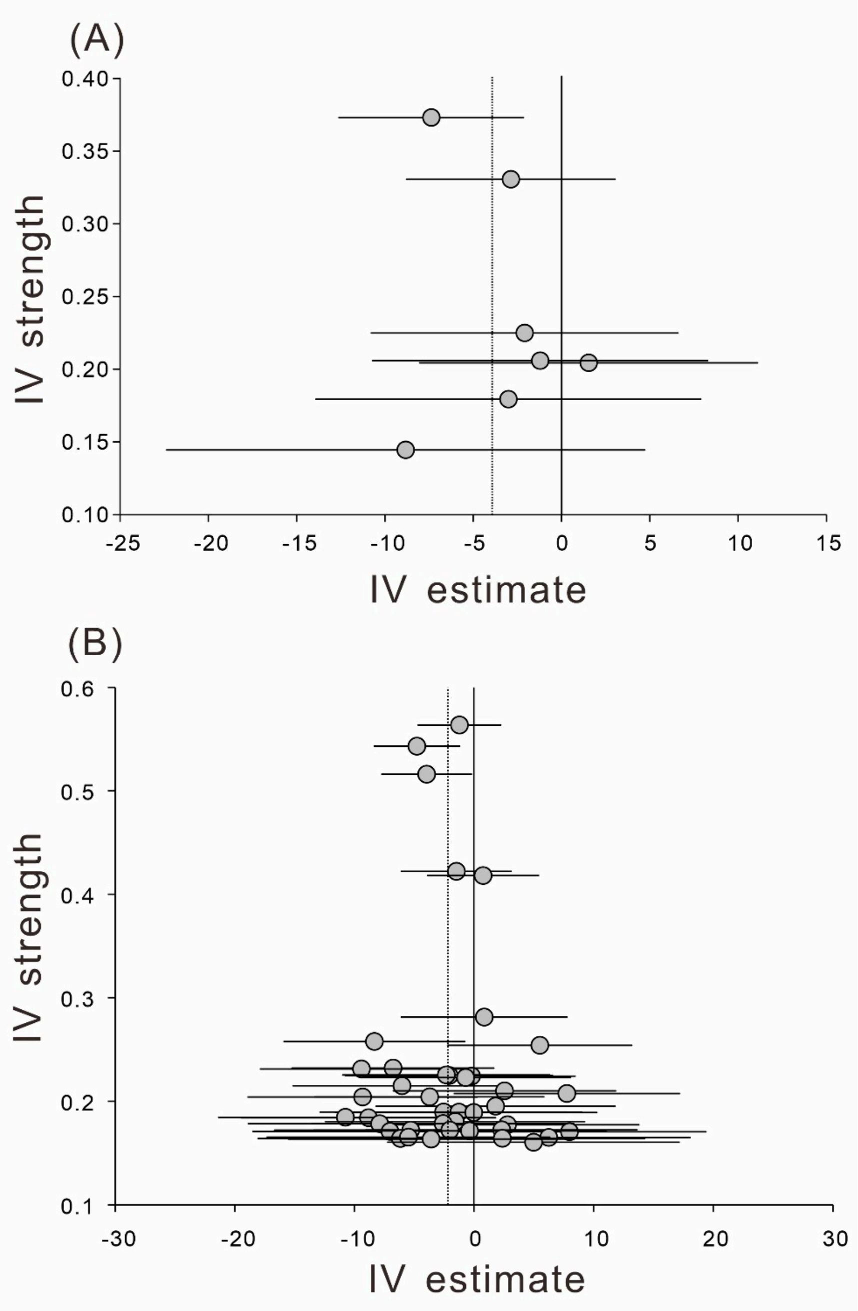

Supplementary Table S22. Estimates of causal effects between the LDL-C levels and DM when using standard Mendelian randomization (MR) sensitivity analysis

	PCSK9 variants on DM				LDL-C GWAS-determined variants on DM			
Analysis Method	Coefficient	S.E.	(95%, CI)	*P* value	Coefficient	S.E.	(95%, CI)	*P* value
Inverse-variance weighted								
Fixed-effect	-3.9309	1.5141	(-7.6357, -0.2261)	9.42×10^{-3}	-2.2050	0.6149	(-3.4476, -0.9623)	3.36×10^{-4}
Random-effects	-3.9309	1.2416	(-6.9691, -0.8927)	1.55×10^{-3}	-2.2050	0.6149	(-3.4476, -0.9623)	3.36×10^{-4}
Egger regression, intercept								
Fixed-effect	-0.0236	0.0099	(-0.0491, 0.0019)	0.0634	-0.0031	0.0039	(-0.0110, 0.0048)	0.4308
Random-effects	-0.0262	0.0122	(-0.0576, 0.0052)	0.0851	-0.0025	0.0041	(-0.0107, 0.0057)	0.5401
Egger regression, slope								
Fixed-effect	-5.3116	1.0983	(-8.1349, -2.4883)	0.0047	-2.3301	0.6374	(-3.6192, -1.0409)	0.0008
Random-effects	-5.3860	1.0362	(-8.0498, -2.7222)	0.0035	-2.1908	0.6436	(-3.4925, -0.8890)	0.0015
Simple median	-2.8732	1.7177	(-7.3760, -1.2130)	0.0949	-2.0270	0.8037	(-3.7083, -0.3769)	0.0117
Weighted median	-2.8732	0.2492	(-3.0119, -3.0119)	< 0.00001	-1.5682	0.6891	(-3.9586, -1.2207)	0.0229

Abbreviation: CI, confidence interval, S.E.: standard error
